# 肺癌筛查研究进展

**DOI:** 10.3779/j.issn.1009-3419.2020.101.37

**Published:** 2020-11-20

**Authors:** 雅喆 管, 萌 任, 冬利 郭, 宇彤 贺

**Affiliations:** 050011 石家庄，河北医科大学第四医院肿瘤研究所 Cancer Institute, Fourth Hospital of Hebei Medical University, Shijiazhuang 050011, China

**Keywords:** 肺肿瘤, 筛查, 成本效益, Lung neoplasms, Screening, Cost-effectiveness

## Abstract

肺癌是世界上最常见的恶性肿瘤，其5年生存率仅为19.7%，严重威胁人类健康。肺癌筛查是降低肺癌死亡率的有效措施，已有的研究证明用低剂量螺旋计算机断层扫描（low-dose computed tomography, LDCT）进行筛查可降低20%的肺癌死亡，目前国际和国内均建议进行肺癌筛查。研究肺癌筛查的发展现状有助于我们明确肺癌的高危人群，探索合理的筛查方案，提高筛查的成本效益，减轻经济负担。因此本文就肺癌筛查现状、肺癌筛查的成本效益以及存在的问题综述如下。

## Abstract (en)

Lung cancer is the most common malignant tumor in the world. The five-year survival rate is 19.7%, which seriously threatens human health. Lung cancer screening is an effective measure to reduce lung cancer mortality. Existing studies have shown that screening with low-dose computed tomography (LDCT) can reduce lung cancer deaths by 20%. Currently, lung cancer screening is recommended internationally and nationally. Studying the development status of lung cancer screening helps us to identify the high-risk groups of lung cancer, explore reasonable screening programs, improve the cost-effectiveness of screening and reduce the economic burden. Therefore, this article summarizes the current status of lung cancer screening, the cost-effectiveness of lung cancer screening and the existing problems as follows.

肺癌是威胁人类生命健康最大的恶性肿瘤之一，据估计，2018年全球范围内约有209万例新发病例，占全部癌症新发病例的11.6%，死亡病例约有176万例，占所有癌症死亡病例的18.4%^[1]^。与多数国家相比，我国肺癌死亡率相对较高，预计从2015年-2030年，我国肺癌死亡率可能会增加40%左右^[2, 3]^。早期肺癌患者症状较轻，临床表现不明显，晚期肺癌患者预后较差，总体5年生存率仅为19.7%，严重影响我国居民的生命健康^[4-6]^。同时，肺癌的医疗费用较高，在诊断后的5年内，每例肺癌患者的直接医疗费用约为40, 650美元，家庭护理人员生产力损失的平均成本（704美元）远高于患者本身（92美元），这给社会和家庭都带来了严重的经济负担^[7]^。因此迫切需要采取有效的防治措施来降低因患肺癌而造成的疾病和经济负担。

早诊早治是提高肺癌患者生存率、减轻经济负担的关键，筛查是早期发现肺癌的有效手段，应用计算机断层扫描（computed tomography, CT）进行筛查可在一定程度上检查出无症状的早期肺癌，降低肺癌死亡率，但仍存在一些问题^[8-11]^。鉴于此，本文对肺癌筛查相关内容展开综述。

## 1 肺癌筛查的研究现状

### 1.1 肺癌重大筛查研究项目

目前比较著名的肺癌筛查研究项目包括国际早期肺癌行动计划（International Early Lung Cancer Program, I-ELCAP）^[12]^、美国国家癌症研究所（National Cancer Institute, NCI）实行的国家肺癌筛查试验（National Lung Screening Trial, NLST）^[9]^、荷兰比利时的肺癌筛查试验（Dutch-Belgian Randomized Lung Cancer Screening Trial, NELSON）^[13]^、意大利的肺癌筛查的研究项目（Multicentric Italian Lung Detection, MILD）^[10]^和德国肺癌筛查干预（Lung cancer Screening Intervention, LUSI）^[11]^等。①I-ELCAP^[12]^是于1992年启动的一项大型非随机对照肺癌筛查项目，此项目共招募了1, 000例无症状志愿者，这些志愿参与者年龄均在60岁或以上，每年至少吸烟10包，并且无癌症史。研究发现，低剂量螺旋计算机断层扫描（low-dose computed tomography, LDCT）筛查可以极大地提高非钙化小结节检出的可能，从而在较早并且可以治愈的阶段检测出肺癌；②NLST^[9]^是于2002年由美国癌症研究所开展实施的一项关于国家肺癌筛查实验的大型随机对照研究项目，该项目将纳入符合要求的人群随机分配到LDCT筛查组和对照组中，经过多年的随访证明了使用LDCT筛查可以降低肺癌的死亡率；③NELSON^[13]^：近期的NELSON进行了最后一轮筛查，其时间间隔为2.5年。在纳入筛查组的7, 915名参与者中，共有5, 279名参与者参加了最后一轮筛查。结果发现与1年筛查间隔相比，2.5年的筛查间隔发现Ⅲb期/Ⅳ期癌症的比例更高，鳞状细胞癌、支气管肺泡癌和小细胞癌的比例也更高。与2年筛查间隔相比，虽然2.5年筛查间隔发现Ⅲb期/Ⅳ期癌症比例高，但无统计学意义。此外，在2.5年的筛查间隔中出现的间隔性癌症比1年和2年筛查间隔出现的更多。这表明2.5年的时间间隔可能太长了，降低了筛查的效果；④MILD筛查计划^[10]^又称为多中心意大利肺部检测，始于2000年在米兰的一项试点研究，并于2005年进行了一项随机试验，共纳入4, 099名参与者，随机分为对照组1, 723名，筛查组2, 376名（1, 190名进行年度LDCT筛查，1, 186名进行两年一次LDCT筛查），2005年-2018年，累计进行了39, 293人年的随访。结果发现，在随访的10年中LDCT组显示出肺癌死亡的风险降低了39%，与对照组相比总体死亡率降低了20%。通过LDCT筛查的收益在筛查的第5年以后有所改善，肺癌死亡率降低了58%，总死亡率降低了32%。MILD试验提供了更多关于LDCT筛查的证据，与NLST试验相比，延长5年以上的筛查可以增强早期检测的益处，实现更大的肺癌死亡率和总体死亡率的降低；⑤LUSI^[11]^是一项针对4, 052例年龄在50岁-69岁之间的长期吸烟者进行的一项随机试验，LDCT筛查组有2, 029名参与者，对照组有2, 023名参与者，平均观察8.8年后发现，男女肺癌死亡率危险比为0.74。按性别进行建模显示，只有女性的肺癌死亡率出现有统计学意义的显著降低。这说明，与男性相比，经LDCT筛查后女性肺癌死亡率降低的幅度更大。

### 1.2 国内外肺癌筛查指南/肺结节处理指南

Fleischner协会于2005年发布了关于肺实性结节的处理指南，于2013年发布了关于肺亚实性结节的处理指南，并在2017年发表了肺部小结节管理指南^[14]^。2011年美国国家综合癌症网络（National Comprehensive Cancer Network, NCCN）发布了肺癌筛查指南，随后每年至少进行一次更新，主要包括肺癌筛查个体的选择、筛查的益处与风险和筛查的成本效益等内容^[15]^。美国癌症协会（American Cancer Society, ACS）于2013年更新了肺癌筛查指南，并于2018年进行了澄清与修改，修改后的指南建议每年对55岁-74岁健康状况良好的成年人进行LDCT肺癌筛查，这些人群包括：过去15年内戒烟并至少有30包/年吸烟史的人群；接受循证戒烟咨询的当前吸烟者；经历了关于使用LDCT筛查潜在益处和危害的知情/共同决策程的人群；并可以使用大量、高质量的肺癌筛查和治疗中心的人群。关于2013年重点关注当前吸烟者戒烟咨询的指导，2018年的更新中指出，戒烟咨询的重要性被更明确地描述为是确定符合肺癌筛查标准的高危人群的重要组成部分，根据减少肺癌死亡率以及平衡筛查利益和伤害的随机对照实验，意图将重点放在一个积极的筛查建议上^[16]^。美国预防服务工作组（U.S. Preventive Services Task Force, USPSTF）发表了肺癌的筛查建议，USPSTF建议对年龄在55岁-80岁、有30包/年吸烟史并且在过去15年内吸烟的所有吸烟者进行年度LDCT筛查。当戒烟时间超过15年并没有出现健康问题时，则建议停止筛查^[17]^。美国胸科协会（American Thoracic Society, ATS）和美国肺脏协会（American Lung Association, ALA）合作发布了指南，旨在帮助克服肺癌筛查计划实施和管理障碍，为开展高质量的肺癌筛查提供了框架^[18]^。2015年，英国胸科学会（British Thoraic Society, BTS）发表了关于英国肺结节的管理指南^[19]^。加拿大放射医师协会（Canadian Association of Radiologists, CAR）根据当前可获得的肺癌筛查最佳实践的文献作为建议发布了指南，该指南对肺癌筛查的纳入和排除标准、筛查的频率和时间以及筛查的潜在影响等问题进行了相关描述^[20]^。我国国家卫健委肺癌早诊早治专家组根据本国的肺癌筛查技术，结合NLST的研究结果，在2009年“农村癌症早诊早治”项目和2012年“城市癌症早诊早治”项目的基础上，分别于2015年和2018年发布了《中国肺癌低剂量螺旋CT筛查指南》。2018年的指南同2015年的指南类似，均包括NLST筛查试验、LDCT筛查系统评价、中国LDCT筛查实践等内容，并在此基础上补充了经LDCT诊断的不同结节或包块的特定临床干预和随访等内容^[21]^。

## 2 肺癌筛查成本效益研究进展

### 2.1 CT筛查费用

考虑筛查项目的经济效益，合理使用卫生资源并且取得较高的成本效益和卫生经济学效果，是肺癌筛查的理想目标。目前大多数肺癌筛查项目常用马尔可夫模型和决策树模型结合起来进行模拟，并用成本效益分析方法来评价肺癌筛查的卫生经济学效果。常用的评价指标有寿命年（life year, LY）、质量调整寿命年（quality adjusted life year, QALY）或增量成本效果比（incremental cost effectiveness ratio, ICER）等。

尽管NLST研究^[9]^已表明经过LDCT筛查可以降低20%的肺癌死亡率，但是针对是否可以通过筛查获得成本效益还需做进一步的讨论。美国NLST实验研究^[9, 22]^表明，进行LDCT筛查，每筛查320例才能预防一例肺癌死亡，每预防一例肺癌死亡大约需额外花费240, 000美元。如果通过NLST的纳入和排除标准，在全国范围内的普通人群中进行筛查费用十分高昂，每年仅CT筛查的费用就超过20亿美元^[23]^。Goulart等^[22]^研究发现，不进行任何筛查的预期治疗费用为28亿美元，当LDCT筛查率达75%时，治疗费用将减少6, 600万美元。并且与不进行筛查相比，经过LDCT筛查，每个人额外花费1, 631美元可获得0.020，1个QALY^[24]^。因此需要客观评价肺癌筛查的成本效益，采用合理的筛查策略，减少肺癌的筛查成本。

### 2.2 LDCT筛查成本效益

#### 2.2.1 高危人群

NCCN指南将肺癌的高危人群定义为年龄在55岁-74岁之间，戒烟 < 15年；年龄 > 50岁，有≥20包/年的吸烟史或其他可以将肺癌风险增加到≥1.3%的风险因素（二手烟除外）的人群，并建议对高危人群进行LDCT筛查^[15]^。

有研究^[24]^对53, 302名参与者进行研究分析发现，和年轻人群相比，对老年人进行LDCT筛查更具成本效益，高年龄组的ICER低于低年龄组（60岁-64岁、65岁-69岁、70岁-74岁每挽救一个QALY分别需花费48, 000美元、54, 000美元和117, 000美元，55岁-59岁每挽救一个QALY需花费152, 000美元）。与曾经吸烟者相比，对目前吸烟者进行LDCT筛查更具有成本效益，曾经吸烟者每挽救一个QALY需花费61, 500美元，当前吸烟者每挽救一个QALY需花费43, 000美元，当前吸烟者的ICER低于曾经的吸烟者。通过模型模拟发现，采用严格吸烟资格的标准，在吸烟率高的国家进行肺癌筛查可能具有成本效益^[25, 26]^。

#### 2.2.2 风险指数

通过应用比NLST的总体纳入标准更为详细的风险目标，可以为每位筛查患者更多地避免早期肺癌死亡^[27]^。Kumar等^[27]^的一项研究中将肺癌的风险因素指数分为十个等级（由低到高为0.1-1），并采用一种新颖的多状态模型来计算每个NLST参与者的预期寿命和收益，研究发现LDCT筛查肺癌的收益随着肺癌死亡风险因素等级的增加而增加，对于风险最低的患者（风险因素指数为0.1），每10, 000人年可预防1.2例的肺癌死亡，而对于风险最高的患者（风险因素指数为1），每10, 000人年则可预防9.5例的肺癌死亡。当用增加的生命年（当肺癌死亡风险因素指数分别为0.1和1时，增加的生命年分别为0.015和0.056）和获得的QALY（当肺癌死亡风险因素指数分别为0.1和1时，获得的QALY分别为0.011和0.028）表示时，随着风险因素的增加，收益也会增加。低风险因素的ICER为每挽救一个QALY花费75, 000美元，高风险因素的ICER为每挽救一个QALY花费53, 000美元。拥有高风险因素的参与者通过LDCT筛查来预防肺癌的死亡所获收益最大，肺癌死亡风险最高的参与者（60%）占筛查死亡率收益的88%^[27, 28]^。使用风险模型来确定最高风险的患者可以使筛查更具成本效益^[29]^。

#### 2.2.3 戒烟干预

CT筛查程序的成本效益将受到筛查参与者戒烟率的强烈影响，美国一项用当时现有的肺癌患者模拟出六个队列的研究^[30]^表明，除非进行筛查会增加戒烟的可能性，否则与不进行年度干预相比，采用LDCT筛查的费用可能要高出100, 000美元/QALY，成本会更昂贵。Villanti等^[31]^使用模型对吸烟人群进行筛查成本效益的评估发现，筛查15年的花费为278亿美元，获得985, 284个QALY，即每挽救一个QALY需花费28, 240美元。当加入戒烟因素后，每挽救一个QALY需16, 198美元-23, 185美元，肺癌筛查的成本效益提高了20%-45%。这表明肺癌筛查应与戒烟干预相结合，戒烟因素的加入可以提高肺癌筛查的成本效益，使筛查变得更加合理^[13, 31]^。

#### 2.2.4 筛查时间间隔

除了筛查人群的选择和戒烟的干预会影响成本效益，时间间隔也会影响LDCT筛查的效益。NELSON试验使用两次筛选之间的间隔（1年、2年和2.5年）来确定最佳筛选频率，较长的时间间隔可能使早期癌症的比例下降，间隔性癌症增加，而较短时间间隔可能会导致成本的增加^[13, 26]^。

有研究^[32, 33]^认为，两年一次的筛查是一种经济有效的策略，在筛查十年后显示与年度筛查相似的肺癌死亡率和总体死亡率。而加拿大的一项研究通过使用模型对三个不同的队列进行模拟发现，虽然每年进行一次CT筛查的额外费用要更高，但是与两年一次的筛查相比，每年一次的筛查可显著降低肺癌死亡率并获得生命年数，因为Ia阶段的生存期比其他阶段高，与年度筛查相比，两年一次的筛查可能更难检测出Ia期的肺癌^[26]^。

筛查的最佳时间间隔尚未确定，与固定间隔相比，更好的选择可能是根据个体患肺癌的风险调整筛查间隔，与开发用于选择筛查对象的模型不同，为了优化筛查间隔而计算个体风险的模型可以基于LDCT图像中检测到的患者特征和病变体征，将患者特征、临床成本与人口统计学数据结合在一起，以评估健康结果和经济影响，针对不同的人群，采用不同筛查间隔^[20, 34-36]^。

关于肺癌筛查的成本效益仍有待进一步研究，选择什么样的筛查人群、如何有效戒烟干预、筛查的频率和间隔时间是多少以及如何准确的辨识良恶性肿瘤显得十分关键。

## 3 肺癌筛查的问题与展望

### 3.1 LDCT筛查

#### 3.1.1 辐射暴露

在目前的筛查实验^[17, 37, 38]^中，肺部每次进行LDCT检查的辐射范围为0.61 mSv-1.5 mSv，常规CT的平均辐射剂量约为7 mSv，而全球平均自然本底辐射水平大约为2.4 mSv，进行一次常规CT检查的辐射剂量约为自然本地辐射的3倍。意大利肺癌筛查试验^[39]^最新分析估计，经过10年LDCT筛查的中位累计辐射剂量男性为9.3 mSv，女性为13.0 mSv，每108例肺癌患者中就有1例由辐射诱发的重大癌症。辐射危害（包括由于累积暴露于辐射而导致的潜在癌症）会因筛查开始时的年龄、患者性别、接受的扫描次数以及暴露于其他电离辐射源（尤其是其他医学影像）而异^[17, 39, 40]^。对于某些患者，其辐射带来的危害可能超过了与筛查相关的潜在益处，因此我们应该关注LDCT筛查引起的辐射问题^[41]^。

#### 3.1.2 假阳性结果

研究^[42]^发现LDCT图像的高假阳性率导致24.2%的筛查参与者需要进一步检查。假阳性结果随肺癌风险的增加而增加，通过肺部CT筛查报告和数据系统来评估筛查假阳性率和肺癌风险的关系发现，随着肺癌风险的增加，筛查结果假阳性的比例由最低风险的12.9%增加到了最高风险的25.9%，同时发现假阳性结果后进行侵入性检查的比例也从最低风险的0.7%增加到了最高风险的2.0%，假阳性结果可能会导致因焦虑而引起的生活质量下降，还可能导致3.76倍的死亡风险^[43, 44]^。

#### 3.1.3 过度诊断

过度诊断也是LDCT肺癌筛查中一个不可忽视的问题，除了筛查的结果为假阳性外，病情进行缓慢的惰性肺癌也可导致过度诊断^[45]^。美国的一项研究使用经过充分验证的微观模型估算了2016年-2030年美国人群进行年度筛查的利弊发现，在筛查出的252, 429例肺癌患者中，有9, 054例被过度诊断，过度诊断率为3.59%，而加拿大的一项研究发现在所有筛查出的肺癌患者中，过度诊断的比例可达12.53%^[26, 46]^。

因此辐射风险、假阳性结果、过度诊断等问题值得我们关注，这些潜在危害进一步强调了以适当和有组织的方式进行筛查的必要性，同时这也给筛查成本效益的评估带来了新的问题，有关LDCT筛查早期肺癌还需要做进一步的研究与讨论。

### 3.2 生物标志物

近年来生物标志物逐渐受到人们关注，主要包括微小RNA（microRNA, miRNA）、甲基化DNA、循环肿瘤DNA（circulating tumor DNA, ctDNA）、自身抗体等^[47]^。虽然越来越多的研究发现血清生物标志物对肺癌的诊断具有一定的辅助价值，但仍有一定的局限性。如miRNA可作为肺癌检测或预后的潜在生物标志物，但与肿瘤的分期和突变体无关^[48, 49]^。血浆和尿液的DNA高甲基化可以作为CT筛查的辅助手段，痰液DNA高甲基化分析可能在临床前疾病的检测中有潜在的价值，但是需要互补的诊断标记来提高敏感性^[50, 51]^。ctDNA作为晚期肿瘤标志物具有一定的价值，然而其在早期肺癌检测中的作用仍不确定，并且成本较高，纳入临床用途比较有限^[52-55]^。肺癌自身抗体具有特异性，但是敏感性较差，仍需要多种肺癌自身抗体联合检测才对肺癌的辅助诊断具有一定的临床价值^[56, 57]^。与LDCT筛查试验相比，大多数生物标志物研究源于相对较小的人群和晚期肺癌的临床实践，用于早期检测肺癌的分子生物标志物目前仍限于研究试验，目前尚无高质量的证据支持或指导这些生物标记物在临床实践中实施^[58, 59]^。作为单一的筛查诊断方法，生物标志物尚不能满足肺癌早期检查的需求。

### 3.3 展望

肺癌筛查利弊共存，通过筛查可以检测及诊断早期肺癌、减轻中晚期疾病负担并提高肺癌患者的生存质量，潜在益处非常大。然而，筛查的成本、辐射暴露、假阳性率高、过度诊断以及生物标志物不敏感等问题仍需考虑。随着科技的进步，人工智能、生物标志物和影像学相结合为肺癌筛查开辟了新途径^[56, 60-62]^，但成本效益和新方法存在的潜在风险不容忽视。构建科学风险评估模型，不断调整完善肺癌筛查模式和指南，设计可以提高成本效益的筛查策略，使肺癌的早期筛查更加精准化、经济化和合理化，在节约成本的同时准确地鉴别出良恶性肿瘤，这将对肺癌的早诊早治、提高患者生存率有着重大的意义。
